# Osteogenic Potential of Osteolforte: Gene and Protein-Level Evaluation in Human Bone Marrow Stromal Cells

**DOI:** 10.3390/cimb47080588

**Published:** 2025-07-24

**Authors:** Da-Sol Kim, Soo-Kyung Bae, Yeon-Ju Kwak, Geum-Joung Youn, Hye-Ock Jang

**Affiliations:** 1 Department of Dental Pharmacology, School of Dentistry, Pusan National University, Yangsan 50612, Republic of Korea; k201797801@pusan.ac.kr (D.-S.K.);; 2 Dental and Life Science Institute, School of Dentistry, Pusan National University, Yangsan-si 50612, Republic of Korea; 3 Research Institute of GH BioFarm, Agricultural Corporation Gagopa-Healing Food, Changwon 51219, Republic of Korea; kyjred@daum.ne (Y.-J.K.);

**Keywords:** Osteolforte, human bone marrow stromal cells (hBMSCs), osteoblast, cell signaling

## Abstract

*Osteolforte*, a compound with potential bone-regenerative properties, was investigated for its effects on human bone marrow stromal cells (hBMSCs). This study aimed to evaluate its impact on cell viability, osteogenic differentiation, and both gene and protein expression using a combination of assays, including CCK-8, Alizarin Red S staining, Quantitative Real-Time PCR (qRT-PCR), and Western blot analysis. The results demonstrated that Osteolforte significantly enhanced osteogenic differentiation in hBMSCs. Alizarin Red S staining revealed increased mineralization, indicating elevated calcium deposition. Gene expression analysis showed an upregulation of key osteogenic markers, including runt-related transcription factor-2 (RUNX-2), collagen type I (COL-1), and bone morphogenetic protein-2 (BMP-2), supporting the role of Osteolforte in promoting osteoblastic activity. In particular, the elevated expression of RUNX-2—a master transcription factor in osteoblast differentiation along with COL-1, a major bone matrix component, and BMP-2, a key bone morphogenetic protein—highlights the compound’s osteogenic potential. In conclusion, Osteolforte enhances early-stage osteogenesis and mineralization in hBMSCs and represents a promising candidate for bone regeneration.

## 1. Introduction

Bone regeneration is a crucial aspect of regenerative medicine, aiming to restore bone integrity and function following injuries, fractures, or degenerative conditions such as osteoporosis. The ability to promote osteogenesis—the formation of new bone tissue—is a primary goal in tissue engineering and regenerative medicine. Various strategies have been employed to enhance bone repair, including the use of biomaterials, growth factors, and stem cell-based therapies. Among these, small-molecule compounds that stimulate osteogenic differentiation in bone marrow stromal cells (BMSCs) have emerged as a promising approach for bone tissue engineering [1]. Mesenchymal stem cells (MSCs) are multipotent progenitor cells capable of differentiating into various mesenchymal lineages, including osteoblasts, chondrocytes, and adipocytes. Their osteogenic differentiation is tightly regulated by numerous signaling pathways and transcription factors, such as runt-related transcription factor-2 (RUNX-2), bone morphogenetic proteins (BMPs), and collagen type I (COL-1), which play key roles in early osteoblastic commitment and matrix mineralization [2]. Understanding the mechanisms for the differentiation of hBMSCs into osteoblasts is essential for developing novel therapeutic agents for bone regeneration.

According to Patent No. RU 2527042 C1, Osteolforte demonstrates osteoinductive potential and is proposed for use in enhancing bone regeneration [3]. Preliminary studies suggest that it enhances osteogenic differentiation, making it a candidate for bone tissue engineering applications. However, its specific mechanisms of action remain largely unexplored. Investigating its effects on cell viability, osteogenic differentiation, and osteogenic marker expression in hBMSCs may offer valuable insights into its biological activity and clinical applicability [4].

Bone is a dynamic tissue that undergoes continuous remodeling, balancing bone formation by osteoblasts and resorption by osteoclasts. In conditions such as osteoporosis, fractures, or bone defects resulting from trauma, infection, or tumor resection, this balance is disrupted, leading to impaired bone healing [5]. Traditional treatments, including bone grafting and synthetic scaffolds, have limitations such as donor site morbidity, immune rejection, and insufficient integration with the host tissue [6]. Hence, alternative strategies that harness the regenerative potential of stem cells and bioactive molecules are gaining traction in the field.

hBMSCs are one of the most extensively studied cell sources for bone regeneration due to their ability to differentiate into osteoblasts under the appropriate stimuli [7]. However, using recombinant proteins and gene therapy approaches comes with safety and cost concerns, highlighting the need for small-molecule compounds that can effectively promote osteogenesis without these weaknesses [8]. The osteogenic differentiation of hBMSCs is controlled by several key markers and signaling pathways. RUNX-2, a transcription factor, is considered the master regulator of osteogenesis, as it controls the expression of essential bone matrix proteins and promotes the transition from mesenchymal stem cells to pre-osteoblasts [7]. Another critical marker is COL-1, which constitutes a major component of the extracellular matrix in bones and provides structural support for mineral deposition. Additionally, BMP-2 plays a vital role in initiating osteoblast differentiation by activating downstream signaling pathways, including the Smad pathway, which drives the expression of osteogenic genes [9]. Interestingly, osteopontin (OPN), a glycoprotein involved in bone remodeling, plays a complex role in osteogenesis, contributing to cell adhesion and mineralization [10]. Investigating the effects of Osteolforte on these molecular markers can provide insights into its osteoinductive potential and reveal any unique regulatory mechanisms it may exert on bone formation. Small-molecule compounds with osteogenic properties have garnered attention due to their ability to target intracellular pathways and enhance osteogenesis efficiently. Unlike recombinant proteins and cell-based therapies, these compounds offer advantages such as ease of administration, cost-effectiveness, and better stability [8,11]. Osteolforte has emerged as a promising candidate, showing potential in preclinical evaluations. This study aims to evaluate the impact of Osteolforte on hBMSC viability, differentiation, and the expression of key osteogenic markers. To comprehensively assess the osteogenic potential of Osteolforte, we employed CCK-8 assays for cytotoxicity, Alizarin Red S staining for mineralization, and qRT-PCR as well as Western blotting for gene and protein expression. The results will contribute to the field of bone tissue engineering and help determine whether Osteolforte can serve as a viable therapeutic agent for bone regeneration.

## 2. Materials and Methods

### 2.1. Cell Culture and Treatments

Human bone marrow-derived mesenchymal stem cells (hBMSCs; PT-2501, Lonza, Basel, Switzerland) were purchased and incubated for specified durations prior to the experimental assessments. According to the manufacturer’s datasheet, the PT-2501 cells were isolated from the bone marrow of a healthy donor. The cells were characterized by flow cytometry based on their surface marker expression: CD105^+^, CD166^+^, CD29^+^, CD90^+^, CD73^+^, and negative for CD133^−^, CD34^−^, and CD45^−^. The cells were cultured in StemMACS™ Media XF supplemented with 1× Anti-Anti (antibiotic–antimycotic solution) at 37 °C in a humidified atmosphere containing 5% CO_2_. Osteolforte (Gagopa Corp., As & Co. Pharm Group LLC, Yekaterinburg, Russia; Patent No. RU 2527042 C1) was applied at concentrations of 0.01, 0.1, and 1 μg/mL.

### 2.2. Osteolforte Preparation

Osteolforte (Gagopa Corp., As & Co. Pharm Group LLC, Yekaterinburg, Russia; Patent No. RU 2527042 C1) is a food additive developed to prevent osteoporosis. It was created through technology transfer from Dr. Sergei in Russia at Gagopa Healing Foods. This is because the key know-how lies in the ratio of the components in the calcium complex formulation, including magnesium, vitamin D3, boron, zinc, selenium, vitamin B6, and calcium. hBMSCs were cultured and treated with Osteolforte (Gagopa Corp.) at concentrations of 0.01, 0.1, and 1 μg/mL. The cells were incubated for specified durations before the assessments.

### 2.3. Cell Proliferation Assay

The in vitro proliferation of hBMSCs was determined using a Cell counting kit-8 (CCK-8, CCK-3000, Dongin Biotech, Seoul, Republic of Korea) assay. The hBMSCs were seeded into 48-well culture plates (30048, SPL, Gyeonggi-do, Republic of Korea) at a density of 1 × 10^4^ cells/well and cultured in StemMACS MSC expansion Media XF (130-101-375, Miltenyi Biote, Bergisch Gladbach, Germany) containing 1X anti-anti (L0010-020, Biowest, Nuaillé, France), and *Osteolforte*. The cells were cultured for 4 days at 37 °C in a 5% CO_2_ incubator. Following this, the CCK-8 assay solution was added and the cells were incubated at 37 °C in a 5% CO_2_ incubator for 2 h. Absorbance was measured using an ELX800 spectrophotometer (BioTek, Winooski, VT, USA) at 450 nm. Each experiment was performed in triplicates.

### 2.4. Osteogenic Differentiation

Osteogenic differentiation was induced by culturing cells for 7–14 days in the osteogenic medium, StemMACS™ OsteoDiff Medium, human (130-091-678, Miltenyi Biote, Bergisch Gladbach, Germany). The calcification of the extracellular matrix was estimated using a 2% Alizarin Red S (ARS) solution (pH 4.3, A-5533, Sigma-Aldrich, St. Louis, MO, USA) for 15 min. To obtain quantitative data, 200 μL of 10% (*w*/*v*) cetylpyridinium chloride (CPC, C-0732, Sigma-Aldrich) and 10 mM of sodium phosphate solution (pH 7.0) were added to the dishes containing the staining solution. The absorbance of the extracted dye was measured at a wavelength of 570 nm.

### 2.5. Osteolimage Mineralization Assay

An OsteoImage™ Mineralization Assay (PA-1503, Lonza, Walkersville, MD, USA) was performed according to the manufacturer’s protocol. Briefly, the cells were cultured in an osteogenic differentiation medium for 7 days. After osteogenic induction, culture plates were washed with PBS and fixed with 70% ethanol for 20 min. Following fixation, the cells were rinsed twice with 1× Wash Buffer and stained with the OsteoImage™ Staining Reagent diluted to 1:100 in the Staining Reagent Dilution Buffer. The staining reaction was carried out at room temperature, protected from light, for 30 min. After incubation, the cells were washed three times with 1× Wash Buffer, with each wash lasting approximately 5 min. Fluorescent signals, corresponding to hydroxyapatite deposition, were measured using a microplate reader at 492 nm excitation and 520 nm emission. The mineralization levels were quantified as relative fluorescence units (RFU), reflecting the extent of calcium phosphate deposition.

### 2.6. Quantitative Real-Time Polymerase Chain Reaction Analysis

Total RNA was isolated using TRIzol reagent (17061, iNtRON Biotechnology Inc., Seongnam, Republic of Korea) according to the manufacturer’s instructions and reverse-transcribed into complementary DNA (cDNA) using a First Strand cDNA Synthesis Kit (K-2041, Bioneer, Daejeon, Republic of Korea). The primer sequences used are shown in Table 1. qRT-PCR was performed using TOPreal™ qPCR 2X PreMIX (RT-500M, SYBR Green with low ROX, Daejeon, Republic of Korea) on an ABI 7500 instrument (Applied Biosystems, Foster City, CA, USA). Data analysis was performed using the _∆∆_Ct method, and the experiments were repeated more than three times.

### 2.7. Western Blot Analysis

Radioimmunoprecipitation assay (RIPA) buffer (Sigma, St. Louis, MO, USA) with a protease inhibitor cocktail (PIC, Roche, Indianapolis, IN, USA) and phosphatase inhibitor was used for cell lysis. The isolated proteins were separated by sodium dodecyl sulfate-polyacrylamide gel electrophoresis (SDS-PAGE) and electro-transferred to PVDF (Millipore, Bedford, MA, USA). The blots were probed with primary antibodies, followed by horseradish peroxidase-conjugated secondary antibodies. The antibodies were detected using an ECL detection kit (Pierce Biotechnology, Rockford, IL, USA) and visualized using an LAS 4000 Luminoimage Analyzer (Fujifilm, Tokyo, Japan). The protein levels were quantified using the National Institute of Health’s ImageJ software (v1.53, NIH, Bethesda, MD, USA). A list of the antibodies used for the Western blot analysis is shown in Table 2.

### 2.8. Statistical Analysis

The results were expressed as the mean ± SEM from at least three independent experiments. The statistical significance between the Osteolforte-treated groups and the untreated control group (0 µg/mL) was determined using Student’s *t*-test, performed with GraphPad Prism version 5.03 (GraphPad Software, San Diego, CA, USA). The values of * *p* < 0.05, ** *p* < 0.01, and *** *p* < 0.001 were considered significant.

## 3. Results

### 3.1. Effect of Osteolforte on Cell Viability and Osteogenic Differentiation in hBMSCs

To investigate both the cytotoxicity and osteogenic potential of Osteolforte in hBMSCs, a two-step in vitro experiment was conducted using concentrations of 0.01, 0.1, and 1 µg/mL.

**Figure 1:**
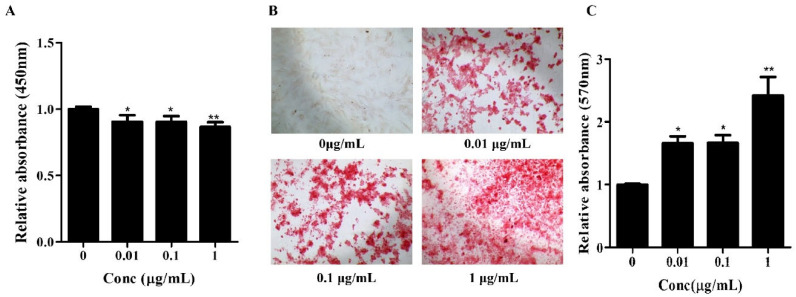
Effects of Osteolforte on cell viability and osteogenic differentiation of BMSCs. (**A**) Cell viability assessed by CCK-8 assay after treatment with Osteolforte at various concentrations (5–100 μg/mL) for 48 h. No significant cytotoxicity was observed across tested concentrations. (**B**) Representative images of Alizarin Red S staining showing calcium deposition in BMSCs treated with Osteolforte at 0, 0.01, 0.1, and 1 μg/mL for 14 days. 100×. The intensity of red staining indicates the extent of osteogenic differentiation. (**C**) Quantification of Alizarin Red S staining by measuring absorbance at 570 nm. Osteolforte significantly enhanced mineralization in a dose-dependent manner. Data are presented as mean ± SEM (*n* = 3). * *p* < 0.05, ** *p* < 0.01 vs. control (0 μg/mL).

First, potential cytotoxic effects were assessed using the CCK-8 assay. hBMSCs were treated with each concentration of Osteolforte for 4 days, and cell viability was subsequently measured. As shown in Figure 1A, there was no significant decrease in cell viability at any concentration when compared to the untreated control group. In all treated groups, the viability remained above 85%, indicating that Osteolforte did not effect cytotoxicity on hBMSCs within this concentration range. These results confirm the safety of Osteolforte treatments and justify their use for further differentiation studies.

Subsequently, the osteogenic potential of Osteolforte was examined in hBMSCs cultured in osteogenic induction medium supplemented with 0.01, 0.1, or 1 µg/mL of Osteolforte for 10 days. Osteogenic differentiation was evaluated by Alizarin Red S (ARS) staining, which detected calcium-rich deposits as a marker of extracellular matrix mineralization. As shown in Figure 1B, Osteolforte treatment led to a notable, concentration-dependent increase in ARS staining, with more intense red staining observed at higher doses. A quantitative analysis of ARS staining was conducted by 10% cetylpyridinium chloride (CPC), and the absorbance was measured at 570 nm (Figure 1C). All concentrations of Osteolforte significantly increased mineral deposition compared with the control, with the most substantial effect observed at 1 µg/mL (** *p* < 0.01).

Together, these findings indicate that Osteolforte is both non-cytotoxic and capable of significantly enhancing osteogenic differentiation in hBMSCs. The observed dose-dependent increase in calcium deposition supports its potential application as a bioactive agent for promoting bone regeneration in regenerative medicine strategies.

### 3.2. Effect of Osteolforte on Osteolimage Mineralization, Quantitative Real-Time PCR and Western Blot Analysis During Osteogenic Differentiation in hBMSCs

To further evaluate the osteoinductive potential of Osteolforte, we assessed early osteogenic activity and the expression of osteogenic markers at both the mRNA and protein levels in hBMSCs.

**Figure 2:**
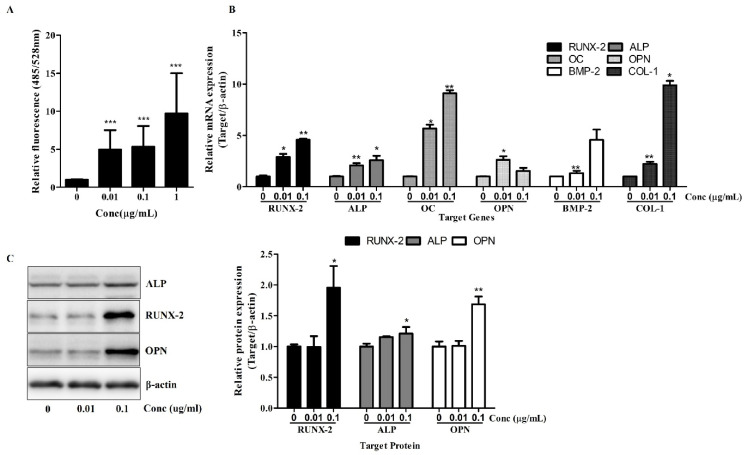
Osteolforte enhances early osteogenic activity and upregulates osteogenic gene and protein expression in hBMSCs. (**A**) ALP activity was quantified using a fluorometric assay (excitation/emission: 485/528 nm) after 7 days of osteogenic induction in the presence of 0.01, 0.1, or 1 µg/mL of Osteolforte. (**B**) Relative mRNA expression of osteogenic markers (RUNX-2, ALP, OC, OPN, BMP-2, and COL-1) was analyzed by qRT-PCR after 7 days of treatment. Expression levels were normalized to β-actin. (**C**) Western blot analysis of RUNX-2, ALP, and OPN protein levels after 7 days of treatment; β-actin was used as a loading control. Densitometric quantification of protein expression is shown in the graph on the right. Data are presented as mean ± SEM (*n* = 3). * *p* < 0.05, ** *p* < 0.01, *** *p* < 0.001 vs. 0 (control).

First, ALP activity, an early marker of osteogenic differentiation, was measured on day 7 using a fluorometric assay. As shown in Figure 2A, Osteolforte treatment significantly increased ALP activity in a dose-dependent manner. Even at the lowest concentration (0.01 µg/mL), ALP activity was markedly elevated compared to the control (*** *p* < 0.001), with the highest activity observed at 1 µg/mL, suggesting enhanced early osteogenic commitment.

Next, a qRT-PCR analysis was conducted to examine the expression of key osteogenic genes. As shown in Figure 2B, treatment with Osteolforte significantly upregulated the mRNA levels of RUNX-2, ALP, Osteocalcin (OC), OPN, BMP-2, and COL-1. Notably, RUNX-2 and OC expression increased by up to 4- to 9-fold at 0.1 µg/mL, while BMP-2 and COL-1 also showed significant upregulation (** *p* < 0.01). These results indicate that Osteolforte activates multiple signaling pathways and transcriptional programs involved in osteoblast differentiation and matrix formation.

A Western blot analysis confirmed the upregulation of osteogenic marker proteins. As shown in Figure 2C, Osteolforte treatment led to an increased expression of RUNX-2, ALP, and OPN proteins, particularly at 0.1 µg/mL. A densitometric analysis revealed a significant increase in ALP, RUNX-2, and OPN levels (* *p* < 0.05 and ** *p* < 0.01, respectively), consistent with the qRT-PCR results.

Collectively, these data demonstrate that Osteolforte not only enhances early osteogenic marker activity but also promotes the transcription and translation of key regulators of osteogenesis in hBMSCs, further supporting its potential as an effective pro-osteogenic agent.

## 4. Discussion

BMSCs have emerged as powerful biological agents in tissue regeneration due to their multi-lineage differentiation potential, immunomodulatory capacity, and trophic effects [12].

This study demonstrates that Osteolforte exerts potent pro-osteogenic effects on hBMSCs without inducing cytotoxicity, thereby supporting its potential as a promising therapeutic agent for bone regeneration.

Our initial cytotoxicity assessment using the CCK-8 assay confirmed the biocompatibility of Osteolforte at all tested concentrations (0.01–1 µg/mL). Cell viability remained above 90% across all groups, indicating that Osteolforte was not toxic for hBMSCs survival.

The osteogenic potential of Osteolforte was demonstrated through multiple complementary assays. Alizarin Red S staining revealed a dose-dependent increase in calcium deposition, with significant mineralization observed at even the lowest dose. This suggests that Osteolforte actively promotes extracellular matrix mineralization, a hallmark of osteoblast maturation. Furthermore, ALP activity, a critical early indicator of osteoblast differentiation [13], was significantly upregulated by Osteolforte treatment. The observed elevation in ALP even at low concentrations highlights the compound’s ability to initiate osteogenic commitment at the early stages of differentiation.

At the molecular level, Osteolforte markedly upregulated the transcription of key osteogenic markers, including RUNX-2, ALP, OC, OPN, BMP-2, and COL-1 [14]. RUNX-2, as a regulator of osteogenesis, showed up to a nine-fold increase, which was further supported by enhanced protein expression in the Western blot analysis. These results suggest that Osteolforte activates multiple osteogenic signaling pathways, promoting both matrix maturation and mineralization. In particular, the concurrent upregulation of BMP-2 and RUNX-2 implies the possible involvement of the canonical BMP/Smad signaling pathway, a well-established regulator of osteoblast differentiation [14]. Although direct pathway validation was not performed in this study, the molecular expression profile observed was consistent with BMP/Smad-mediated osteogenic responses. Future studies are needed to confirm this mechanism through functional assays.

Notably, the protein expression levels of OPN and ALP also increased in a pattern consistent with the gene expression data, validating the translational relevance of the observed transcriptional changes. Interestingly, while OPN gene expression showed a dose-dependent decrease in the qRT-PCR analysis, its protein level was elevated in the Western blot results, suggesting potential post-transcriptional regulation. Therefore, further studies are required to clarify the mechanisms underlying this discrepancy.

The ability of Osteolforte to enhance osteogenic differentiation at both the early and late stages suggests its promise as an adjunct in regenerative therapies, particularly for treating large bone defects or impaired bone healing. The concentration-dependent effects observed further support its therapeutic relevance.

However, as this study was limited to an in vitro analysis using hBMSCs, which, although widely accepted as a model for bone biology, does not fully replicate in in vivo conditions, further preclinical and clinical studies are required to evaluate its pharmacokinetics, bioavailability, and long-term safety.

In conclusion, our findings highlight Osteolforte as a non-cytotoxic, osteoinductive compound that promotes the osteogenic differentiation of hBMSCs through the upregulation of key genetic and protein markers. These results lay the groundwork for further preclinical development of Osteolforte as a candidate agent for bone tissue engineering and regenerative medicine.

## 5. Conclusions

Bone regeneration remains a major challenge in regenerative medicine, requiring new strategies to enhance osteogenesis. Osteolforte, a newly identified compound, has shown potential in promoting hBMSC differentiation into osteoblasts, though its mechanism of action is not yet fully understood. This study evaluates the effects of Osteolforte on key osteogenic markers—including RUNX-2, COL-1, BMP-2, and OPN—to determine its therapeutic potential. The results may provide insights into its biological activity and support future applications in bone repair.

## Figures and Tables

**Table 1:** Quantitative Real-time PCR primer sequences.

Name	Sequences (5′ → 3′)	
ALP	F	GACCTCCTCGGAAGACACTC
	R	TGAAGGGCTTCTTGTCTGTG
RUNX-2	F	GGTTAATCTCCGCAGGTCACT
	R	CACTGTGCTGAAGAGGCTGTT
OC	F	GCAGCGAGGTAGTGAAGAGAC
	R	AGCAGAGCGACACCCTAGA
OPN	F	CAAGACAGTGCCCAAGATAC
	R	TTCCCTCATCGTCCAACT
BMP2	F	ACCCGCTGTCTTCTAGCGT
	R	CTCAGGACCTCGTCAGAGGG
COL-1	F	CAGCCGCTTCACCTACAGC
	R	TTTTGTATTCAATCACTGTCTTGCC
β-actin	F	GGCACCCAGCACAATGAAG
	R	TGCGGTGGACGATGGAGG

**Table 2:** Western Blot Antibody List.

Protein	Catalog Number
ALP (B-10)	sc-365765, Santa Cruz Biotechnology (Dallas, TX, USA)
OPN (Osteopontin)	#PA5-16821, Invitrogen (Carlsbad, CA, USA)
RUNX-2 (D1L7F)	#12556, Cell Signaling (Danvers, MA, USA)
β-actin	sc-47778, Santa Cruz Biotechnology
Goat anti-rabbit IgG-HRP	sc-2004, Santa Cruz Biotechnology
Goat anti-mouse IgG-HRP	sc-2005, Santa Cruz Biotechnology

## Data Availability

Data is contained within the article.

## Abbreviations

hBMSCs	human bone marrow stromal cells
GSK-3β	glycogen synthase kinase 3 beta
HLA	human leukocyte antigen
DMSO	Dimethyl sulfoxide
CCK-8	Cell counting kit-8
ARS	Alizarin Red S
CPC	Cetylpyridinium chloride
qRT-PCR	Quantitative real-time polymerase chain reactin
SEM	standard error of the mean
